# Bone Health and Anti-Osteoporotic Medication Eligibility in Postmenopausal Women Undergoing Bariatric Surgery: The Impact of Age and Modifiable Risk Factors

**DOI:** 10.1007/s00223-026-01484-z

**Published:** 2026-01-31

**Authors:** Line Abdulghani, Hélène Verkindt, Laurine Cadart, Cécile Philippoteaux, Robert Caiazzo, Julien Paccou

**Affiliations:** 1 Rheumatology Department, CHU Lille, 59000 Lille, France; 2 General Endocrine Surgery, Lille University Hospital Chu Lille, Egid-Umr 1190, Translational, Research Laboratory for Diabetes, Lille University, CHU Lille, Lille, France; 3 Biostatistics Department, CHU Lille, 59000 Lille, France; 4 Rheumatology Department, ULR 2694 - METRICS, Lille University Hospital, Lille University, 59000 Lille, France; 5 Rheumatology Department, MABlab ULR 4490, University Hospital of Lille, Univ. Lille, CHU Lille, 59000 Lille, France

**Keywords:** Bariatric surgery, Obesity, Osteoporosis, Fractures, Vitamin D, Post-menopausal women, Calcium intake, Parathyroid hormone, Physical activity

## Abstract

**Introduction:**

Postoperative complications after metabolic and bariatric surgery (MBS) increase with age, yet data on bone health in older postmenopausal women remain limited. The 2022 European Calcified Tissue Society (ECTS) position statement recommend anti-osteoporotic medication (AOM) for patients with a T-score ≤ −2 and/or a fragility fracture within the past two years. This study evaluated the prevalence of AOM eligibility according to age (< 60 vs. ≥ 60 years) in postmenopausal women and identified associated risk factors.

**Materials and Methods:**

We conducted a cross-sectional, observational, single-center study at Lille University Hospital including postmenopausal women referred for bone health evaluation before or after MBS. AOM eligibility was defined according to the 2022 ECTS criteria.

**Results:**

Among 306 postmenopausal women, 173 were < 60 years (group 1) and 133 were ≥ 60 years (group 2). Overall, 69 patients (22.5%) were eligible for AOM, with a significantly higher prevalence in women ≥ 60 years (30.1% vs. 16.8%, *p* = 0.006). In multivariate analysis, independent predictors of AOM eligibility were age ≥ 60 years (OR = 2.34, 95% CI: 1.19–4.61), active smoking (OR = 2.98, 95% CI: 1.27–7.02), reduced appendicular lean mass index (ALMI < 5.5 kg/m^2^; OR = 3.72, 95% CI: 1.78–7.78), and elevated iPTH (OR = 1.15, 95% CI: 1.05–1.25 per 10 ng/mL increase).

**Conclusion:**

Nearly one in five postmenopausal women undergoing MBS were eligible for AOM based on the ECTS position statement, with prevalence doubling after 60 years (OR = 2.34). Active smoking, secondary hyperparathyroidism, and low ALMI were key independent risk factors. These findings underscore the importance of systematic bone health assessment and early implementation of lifestyle interventions, alongside AOM, to reduce fracture risk in this vulnerable population.

## Introduction

Obesity is a major public health concern, associated with cardiovascular disease, type 2 diabetes mellitus (T2DM), sleep apnea, hypertension, and certain cancers. Its prevalence continues to rise worldwide, affecting 16% of adults in 2022 [1, 2]. Metabolic and bariatric surgery (MBS) is considered the most effective treatment for severe obesity, and its use has increased dramatically over the past two decades. In France, procedures reached 72 per 100,000 inhabitants in 2018, with a notable rise among patients over 65 years of age [3–6].

While MBS improves quality of life and weight-related comorbidities [7], it can have deleterious effects on bone health. A meta-analysis reported a 29% increased fracture risk in patients undergoing MBS compared with controls, particularly after malabsorptive procedures such as Roux-en-Y gastric bypass (RYGB) or biliopancreatic diversion with duodenal switch (BPD-DS) [8, 9].

In 2022, the European Calcified Tissue Society (ECTS) recommended systematic bone health assessment in postmenopausal women and men ≥ 50 years, with eligibility for anti-osteoporotic medication (AOM) based on recent fragility fractures, low bone mineral density (BMD), or high FRAX® scores [10].

To date, only one retrospective study (Courtalin et al*.*) has applied the ECTS criteria in the MBS setting, identifying 19.2% of eligible patients but including a heterogeneous population of men and women [11]. The French National Authority for Health (HAS) has also highlighted increased postoperative morbidity and mortality beyond age 60 [3], yet without considering skeletal fragility.

Postmenopausal women represent a subgroup at particularly high risk due to the combined effects of age, estrogen deficiency, and post-surgical metabolic alterations. Better characterization of fracture risk in older postmenopausal women is therefore needed. In this study, we assessed the prevalence of eligibility for AOM based on the 2022 ECTS criteria among postmenopausal women undergoing bone health evaluation as part of a MBS care pathway. Our primary objective was to compare prevalence between women < 60 and ≥ 60 years, and secondarily to identify additional risk factors independently associated with AOM eligibility.

## Patients and Methods

### Study Design

A Single-center, descriptive, cross-sectional study was conducted at Lille University Hospital.

**Fig. 1:**
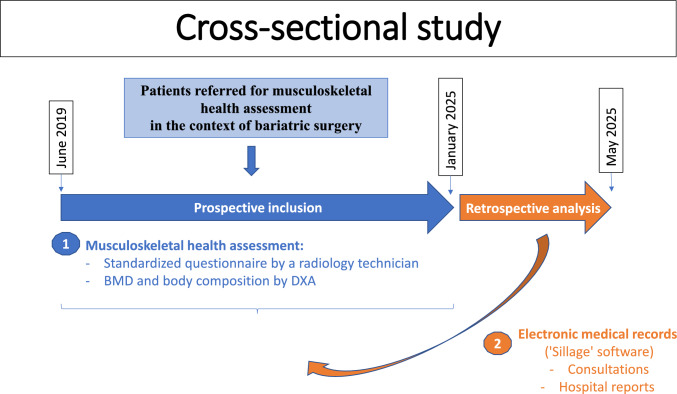
Study design

The patients were prospectively referred between June 2019 and January 2025. Data were analyzed retrospectively. The study included patients routinely referred to the Rheumatology Department of Lille University Hospital for bone assessment in the context of bariatric surgery, either prior to the procedure or during postoperative follow-up (Fig. 1). This study was approved by institutional review board.

### Study Population

This study was conducted exclusively among postmenopausal women. Participants were included if they had been referred for one of three indications: an initial evaluation prior to first-time bariatric surgery (FIRST), postoperative follow-up after bariatric surgery (FOLLOW-UP), or evaluation before undergoing a subsequent bariatric procedure (RE-DO).

The assessment included BMD measurement using dual-energy X-ray absorptiometry (DXA) at the time of inclusion. The vast majority of BMD scans (> 95%) were performed at Lille University Hospital on the same machine (HOLOGIC Discovery A S/N 81360). In rare cases, scans conducted in private practices were also collected.

The criteria used for obesity and bariatric surgery indication followed national guidelines, namely: body mass index (BMI) ≥ 40 kg/m^2^, or BMI ≥ 35 kg/m^2^ with associated comorbidities (such as type 2 diabetes, hypertension, obstructive sleep apnea, or dyslipidemia).

All bariatric surgery procedures were eligible for inclusion: sleeve gastrectomy (SG), RYGB, adjustable gastric banding (AGB), BPD-DS, single anastomosis duodeno-ileal bypass with sleeve gastrectomy (SADI), Vertical Gastric Clip, and vertical banded gastroplasty. Surgical procedures could have been performed either at Lille University Hospital or in other healthcare facilities.

Premenopausal women and male patients were excluded from this study. Additionally, patients who did not undergo BMD assessment were not included in this study.

### Data Sources

Data were retrospectively collected using a variety of sources: (i) a standardized questionnaire completed by a radiology technician during the DXA assessment when the procedure was performed at Lille University Hospital, and (ii) electronic medical records accessed via the 'Sillage' software of Lille University Hospital: results of BMD and body composition by DXA, rheumatology consultations, follow-up consultations, or day hospital reports from the department of general surgery and endocrinology.

### Data Collection

The collected data included the following parameters.

- Demographic characteristics: age at inclusion, weight, height, BMI, and age at menopause,
- Perioperative status: classification of preoperative (FIRST or RE-DO) or postoperative evaluation (FOLLOW-UP),
- Medical and surgical history including current medications,
- Osteoporosis clinical risk factors: personal history of low-energy fractures, first-degree family history of hip fractures, early menopause (< 45 years), chronic use of proton pump inhibitors (PPIs), prolonged corticosteroid therapy (≥ 3 months at a dosage ≥ 7.5 mg/day prednisone equivalent), current or past smoking, current or past excessive alcohol consumption (based on the mention of excessive alcohol consumption or alcoholism, or by a reported intake ≥ 3 units of alcohol per day), and history of chronic inflammatory rheumatic diseases, T2DM, or hyperthyroidism,
- Opportunistic imaging analysis, when available, including thoracolumbar spine radiographs or abdominopelvic CT scans, aimed at identifying vertebral fractures,
- History of bariatric surgery, specifying the type of procedure performed and the time interval (in years) between the first surgery and the DXA evaluation. The scheduled surgical procedure was recorded for patients who had not yet undergone surgery.

### Biological Data

The following biological parameters were collected if available: 25(OH) vitamin D, intact parathyroid hormone (iPTH), calcium, creatinine, estimated glomerular filtration rate (eGFR) using the CKD-EPI formula, glycemia, glycated hemoglobin (HbA1c) and C-reactive protein (CRP).

### BMD Assessment

Surgical status (preoperative or postoperative) at the time of DXA evaluation was systematically documented. BMD was expressed in grams per square centimeter (g/cm^2^) of hydroxyapatite and analyzed using T-score values at the lumbar spine (L1–L4), femoral neck, and total hip. Osteopenia was defined as a T-score between − 2.5 and − 1.0 at any sites. Osteoporosis was defined as a T-score ≤  − 2.5 in accordance with the WHO diagnostic criteria. Additionally, the presence of a T-score ≤  − 2.0 at any site was specifically identified.

### Body Composition Assessment

Measurements included: Total body fat (TBF) expressed in both percentage (%) and kilograms (kg); Visceral adipose tissue (VAT) area measured in square centimeters (cm^2^); Fat mass index (FMI) expressed in kilograms per square meter (kg/m^2^); Total lean mass (TLM) in kilograms (kg), and appendicular lean mass (ALM) in kilograms (kg); Appendicular lean mass index (ALMI) in kilograms per square meter (kg/m^2^). Low lean mass was defined as an ALMI of ≤ 5.5 kg/m^2^.

### Study Outcomes

#### Primary Outcome

To compare the prevalence of eligibility for AOM between two groups of postmenopausal women: those aged < 60 years (group 1) and those aged ≥ 60 years (group 2). Eligibility for AOM was defined according to the 2022 ECTS criteria [10]: (i) a personal history of fragility fracture within the past two years and (ii) T-score ≤ -2 at any measurement site.

We did not include the third criterion based on the FRAX® score, as it proved to be less discriminative compared to the other two criteria [11].

#### Secondary Outcomes

- Comparison of the prevalence of personal history of fragility fractures within the past two years between the two groups,
- Comparing the two groups, the prevalence of a T-score ≤  − 2.0 at any measurement site,
- To identify risk factors associated with eligibility for AOM in the overall study population.

All candidate risk factors for AOM eligibility were preselected based on their relevance for osteoporosis. These included demographic variables (age group < 60 vs ≥ 60 years, BMI, ALMI), nutritional/metabolic status (expert-diagnosed chronic malnutrition, T2DM, history of malabsorptive surgery such as RYGB, BPD-DS, and SADI), surgical pathway (FIRST, FOLLOW-UP, or RE-DO) and time since first MBS, lifestyle factors (smoking, excessive alcohol use, PPI therapy), as well as biochemical parameters (iPTH and 25(OH)D levels at the time of bone evaluation).

### Statistical Analysis

Categorical variables were expressed as frequencies and percentages. Quantitative variables were expressed as mean and standard deviation or as median and interquartile range [IQR] in the case of non-Gaussian distributions. The normality of distributions was verified graphically and using the Shapiro–Wilk test.

The two age groups (< 60 years and ≥ 60 years) were compared in terms of general characteristics, history of bariatric procedures, BMD, body composition, and biological data using a Chi-square test (or Fisher’s exact test in case of expected frequency < 5) for categorical variables, or using Student’s t-test (or Mann Whitney test in case of non-normal distribution) for quantitative variables.

The association between eligibility for AOM and the two age groups was analyzed using a chi-square test. The association of each of the components of this criterion with age group was also studied separately using a chi-square test.

The identification of risk factors for AOM eligibility was performed through univariate logistic regression analyses. The log-linearity assumption for each quantitative factor was assessed by using cubic spline functions. Variables that did not satisfy this assumption were categorized. Factors with a p-value of less than 0.10 were included in a multivariate logistic regression model. Collinearity between the parameters was checked using the variance inflation factor (VIF). Odds ratios (ORs) and 95% confidence intervals (CIs) were reported as measures of association.

Statistical testing was conducted at a 2-tailed α-level of 0.05. Data were analyzed using SAS (release 9.4 (SAS Institute, Cary, NC, USA).

## Results

### Characteristics of the Study Population

Between June 2019 and January 2025, 442 patients were referred for bone health assessment including 306 postmenopausal women. Among 306 postmenopausal women (mean age 58.6 ± 6.3 years), 173 were < 60 years (group 1) and 133 ≥ 60 years (group 2). Mean BMI was 35.3 ± 8.3 kg/m^2^, with no group difference. Hypertension, T2DM, and chronic kidney disease were significantly more frequent in group 2, whereas medication use did not differ between groups (Table 1).

**Table 1:** Characteristics and prevalence of osteoporotic risk factors in the study population

	*N*	Overall	*N*	Group 1 < 60 years old	*N*	Group 2 ≥ 60 years old	*P*-value
		(*N* = 306)		(*N* = 173)		(*N* = 133)	
Age (years)	306	58.6 ± 6.3	173	54.2 ± 4.3	133	64.2 ± 3.4	–
Weight (kg)	306	91.7 ± 21.3	173	91.0 ± 22.3	133	92.6 ± 19.9	0.50
Height (cm)	306	161.2 ± 6.6	173	161.8 ± 6.4	133	160.3 ± 6.7	0.050
Body mass index (kg/m^2^)	306	35.3 ± 8.3	173	34.8 ± 8.5	133	36.1 ± 8.0	0.16
o < 30		76 (24.8)		48 (27.7)		28 (21.1)	0.70
o 30–34.9		70 (22.9)		38 (22.0)		32 (24.1)	
o 35–39.9		80 (26.1)		44 (25.4)		36 (27.1)	
o 40–50		67 (21.9)		37 (21.4)		30 (22.6)	
o > 50		13 (4.2)		6 (3.5)		7 (5.3)	
*Comorbidities*							
High blood pressure	302	191 (63.2)	170	97 (57.1)	132	94 (71.2)	0.011
Obstructive sleep apnea	304	174 (57.2)	172	104 (60.5)	132	70 (53.0)	0.19
Diabetes	306	101 (33.0)	173	48 (27.7)	133	53 (39.8)	0.026
Thyroid dysfunction	303	64 (21.1)	171	33 (19.3)	132	31 (23.5)	0.38
Dyslipidemia	304	104 (34.2)	172	56 (32.6)	132	48 (36.4)	0.49
Chronic kidney disease	304	16 (5.3)	172	5 (2.9)	132	11 (8.3)	0.036
*Treatments*							
Antihypertensive drugs	299	148 (49.5)	170	83 (48.8)	129	65 (50.4)	0.79
Oral antidiabetic medications	300	75 (25.0)	171	37 (21.6)	129	38 (29.5)	0.12
Lipid-lowering drugs	300	72 (24.0)	171	43 (25.1)	129	29 (22.5)	0.59
Proton pump inhibitors	300	109 (36.3)	171	55 (32.2)	129	54 (41.9)	0.084
Vitamin D supplements	296	143 (48.3)	170	83 (48.8)	126	60 (47.6)	0.84
Calcium supplements	291	96 (33.0)	167	56 (33.5)	124	40 (32.3)	0.82
Levothyroxine	300	53 (17.7)	171	29 (17.0)	129	24 (18.6)	0.71
Osteoporotic risk factors							
*Excessive alcohol consumption*							
o Current	305	32 (10.5)	172	20 (11.6)	133	12 (9.0)	0.39
o Former		8 (2.6)		6 (3.5)		2 (1.5)	
*Smoking*							
o Current	304	40 (13.2)	171	33 (19.3)	133	7 (5.3)	< 0.001
o Former		88 (28.9)		55 (32.2)		33 (24.8)	
Early menopause	272	57 (21.0)	149	33 (22.1)	123	24 (19.5)	0.60
Family history of hip fracture	303	14 (4.6)	170	7 (4.1)	133	7 (5.3)	0.64
Previous use of corticosteroids	299	18 (6.0)	169	8 (4.7)	130	10 (7.7)	0.29
Chronic inflammatory rheumatism diseases	300	14 (4.7)	129	8 (4.7)	131	6 (4.6)	0.95

Values are presented as mean ± standard deviation or number (percentage)

In this cohort, several risk factors for osteoporosis were identified (Table 1). Early menopause was reported in 21.0% of patients. Smoking was common in this cohort, with 13.2% identified as current smokers and 28.9% as former smokers. Notably, current smoking was significantly less common in the group 2 (5.3% versus 19.3%, *p* = 0.001). Excessive alcohol consumption was observed in 10.5% of the population. Other risk factors for osteoporosis were less common.

**Fig. 2:**
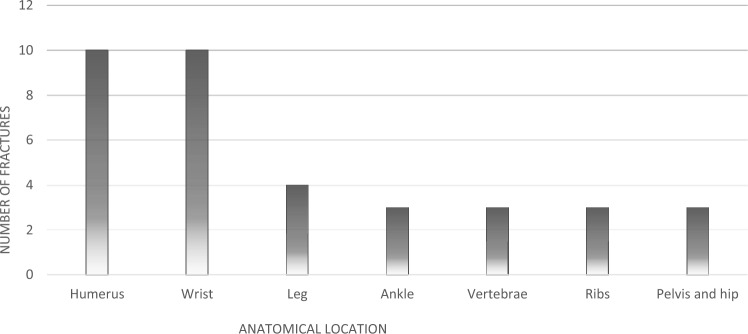
Graphical representation of fragility fracture distribution by anatomical site within the past two years

Thirty-one women (10.3%) reported at least one fragility fracture within the past two years, without significant group difference. Most fractures involved the upper limb, followed by lower limb and vertebral sites. Figure 2 illustrates the distribution of these fractures by anatomical site.

Table 2 summarizes the history of bariatric surgery. In terms of bone health assessment timing, 62.4% of patients were in the FOLLOW-UP group, 22.6% in the FIRST group, and 15.0% in the RE-DO group. There was no significant difference in the timing of bone health assessment between the two age groups (*p* = 0.93). A total of 236 women (77.4%) had a history of at least one bariatric surgery, with similar rates between the two age groups (*p* = 0.97). Among the study participants, 54.9% had undergone malabsorptive procedures (*n* = 168) although these types of procedure were less common in the group 2 (48.9% versus 59.5%, *p* = 0.06). The median interval between the last MBS and the bone health assessment was five years [IQR: 1–11], and this was consistent across both groups (p = 0.28). Similarly, the number of bariatric procedures did not significantly differ between the age groups (p = 0.64).

**Table 2:** History of bariatric surgery

	Overall	Group 1 < 60 years old	Group 2 ≥ 60 years old	*P*-value
	(*N* = 306)	(*N* = 173)	(*N* = 133)	
*Bone assessment status*				
o Assessed before the first surgery	69 (22.6)	38 (22.0)	31 (23.3)	0.93
o Assessed during follow-up	191 (62.4)	108 (62.4)	83 (62.4)	
o Assessed before another surgery	46 (15.0)	27 (15.6)	19 (14.3)	
History of bariatric surgery	236 (77.4)	134 (77.5)	102 (77.3)	0.97
History of malabsorptive surgery	168 (54.9)	103 (59.5)	65 (48.9)	0.063
Number of surgery*	1 [1, 2]	1 [1, 2]	1 [1–1]	0.64
Time between last surgery and bone health assessment* (years)	5 [1–11]	5 [1–11]	6 [1–12]	0.28

^*^ In case of history of bariatric surgery (*N* = 236). For patients without history of bariatric surgery, the number of surgery and the time between first surgery and bone health assessment were considered at 0

Values are presented as number (percentage) or median [25th–75th percentiles]

The median iPTH level was 48 pg/ml [35–66] and the mean 25(OH) vitamin D levels was 25.8 ng/ml (± 9.8). Over 25% of patients had secondary hyperparathyroidism (iPTH > 66 pg/mL). Group 2 had a significantly higher serum calcium level compared to group 1 (*p* = 0.030), as well as a significantly lower eGFR (*p* = 0.003).

### Bone Mineral Density and Body Composition by DXA

BMD was significantly lower in women ≥ 60 years at all sites, with osteoporosis more prevalent (18.0% vs 5.2%, *p* < 0.001) and normal BMD less frequent (37.6% vs 53.8%, *p* = 0.005). A T-score ≤ –2 was observed in 26.3% of group 2 versus 11.6% of group 1 (*p* < 0.001). Body composition did not differ between groups (Table 3).

**Table 3:** Bone mineral density and body composition data

	N	Overall	N	Group 1 < 60 years old	N	Group 2 ≥ 60 years old	*P*-value
		(*N* = 306)		(*N* = 173)		(*N* = 133)	
*BMD data*							
*All sites*							
Normal	306	143 (46.7)	173	93 (53.8)	133	50 (37.6)	0.005
Osteoporosis	306	33 (10.8)	173	9 (5.2)	133	24 (18.0)	< 0.001
Osteopenia	306	129 (42.2)	173	70 (40.5)	133	59 (44.4)	0.49
T score ≤ -2	306	55 (18.0)	173	20 (11.6)	133	35 (26.3)	< 0.001
*Total hip*							
BMD (g/cm^2^)	294	0.925 ± 0.142	167	0.955 ± 0.132	127	0.885 ± 0.146	< 0.001
T-score (SD)	297	−0.1 ± 1.1	169	0.1 ± 1.0	128	−0.4 ± 1.1	< 0.001
*Femoral neck*							
BMD (g/cm^2^)	296	0.762 ± 0.122	167	0.790 ± 0.120	129	0.726 ± 0.114	< 0.001
T-score (SD)	299	−0.8 ± 1.0	169	−0.6 ± 1.0	130	−1.2 ± 0.9	< 0.001
*Lumbar spine*							
BMD (g/cm^2^)	300	1.004 ± 0.175	169	1.023 ± 0.168	131	0.979 ± 0.181	0.031
T-score (SD)	303	−0.1 ± 1.5	171	0.1 ± 1.4	132	−0.3 ± 1.7	0.026
*Body composition*							
TBF (kg)	293	45.0 ± 15.1	168	44.6 ± 15.9	125	45.5 ± 14.0	0.61
FMI (kg/m^2^)	296	17.2 [13.9–21.3]	171	16.8 [13.5–21.1]	125	17.5 [14.6–21.8]	0.13
VAT (cm^2^)	296	212.5 ± 93.3	170	203.5 ± 93.6	126	224.7 ± 92.0	0.053
TLM (kg)	294	42.6 ± 7.2	169	42.5 ± 7.6	125	42.8 ± 6.6	0.70
ALMI (kg/m^2^)	297	6.6 ± 1.4	171	6.6 ± 1.4	126	6.6 ± 1.3	0.92

Values are presented as number (percentage), median [25th–75th percentiles] or mean ± standard deviation. ALMI: appendicular lean mass index, BMD: bone mineral density, FMI: fat mass index, TBF: total body fat, TLM: total lean mass, VAT: visceral adipose tissue

### Prevalence of Eligibility for AOM According to ECTS Recommendations

The prevalence of eligibility for AOM was significantly higher in group 2 (30.1% (*n* = 40/133) versus 16.8% (*n* = 29/173), *p* = 0.006).

Regarding the individual components of the composite outcome:

- The prevalence of recent fragility fractures was not significantly different between age groups (8.2% in group 1 (*n* = 14/173) versus 13.0% in group 2 (*n* = 17/133), *p* = 0.17).
- A significantly greater prevalence of patients in group 2 had a T-score ≤ –2 (26.3% (*n* = 35/133) versus 11.6% (*n* = 20/173), *p* < 0.001), especially at the femoral neck (*n* = 40/55).

### Risk Factors Associated with Eligibility for AOM

In the univariate analysis, several variables showed a significant association with the eligibility for AOM: age group (*p* = 0.006), lower BMI (*p* = 0.002), lower ALMI (*p* =  < 0.001), higher iPTH (*p* = 0.010), current smoking (*p* = 0.019) and the time between first surgery and BMD assessment (*p* = 0.009) in case of history of MBS. The multivariate analysis identified several factors that were independently associated with eligibility for AOM (Table 4). Individuals aged ≥ 60 years demonstrated more than a twofold increased likelihood of being eligible (OR 2.34, 95% CI 1.19–4.61, *p* = 0.014). Current smoking was also strongly associated with eligibility (OR 2.98, 95% CI 1.27–7.02, *p* = 0.012). In addition, low ALMI (≤ 5.5 kg/m^2^ versus > 5.5 kg/m^2^) markedly increased the odds of being eligible for AOM (OR 3.72, 95% CI 1.78–7.78, *p* = 0.001). Finally, higher iPTH levels were associated with eligibility, with an OR of 1.15 for every 10 ng/ml increase (95% CI 1.05–1.25).

**Table 4:** Results for uni- and multivariate logistic regression analysis of risk factors for eligibility to anti-osteoporosis medications.

	Not eligible for AOM (*N* = 237)	Eligible for AOM (*N* = 69)	Univariate analysis OR [95%CI]	*P*-value	Multivariate analysis OR [95%CI]	*P*-value
*Group*						
o 1 (< 60 years old)	144 (60.8)	29 (42.0)	ref	0.006	ref	0.014
o 2 (≥ 60 years old)	93 (39.2)	40 (58.0)	2.14 [1.24–3.68]		2.34 [1.19–4.61]	
*Bone status assessment*						
o 1 (Initial evaluation prior to first-time bariatric surgery)	60 (25.3)	9 (13.0)	Ref	0.064	ref	0.58
o 2 (Postoperative follow-up after bariatric surgery)	140 (59.1)	51 (73.9)	2.43 [1.12–5.25]		1.21 [0.43–3.42]	
o 3 (Evaluation before another bariatric surgical procedure RE-DO)	37 (15.6)	9 (13.0)	1.62 [0.59–4.46]		0.75 [0.19–2.96]	
*Body mass index (kg/m*^*2*^*)*						
o < 30	52 (21.9)	24 (34.8)	ref	0.002		
o 30–34.9	47 (19.8)	23 (33.3)	1.06 [0.53–2.12]			
o 35–39.9	72 (30.4)	8 (11.6)	0.24 [0.10–0.58]			
o ≥ 40	66 (27.9)	14 (20.3)	0.46 [0.22–0.98]			
*History of malabsorptive surgery*						
o No	111 (46.8)	27 (39.1)	ref	0.26		
o Yes	126 (53.2)	42 (60.9)	1.37 [0.79–2.37]			
Time between last surgery and bone mineral density assessment (years)*	5 [0—11]	8 [2–14]	1.05 [1.01–1.09]	0.009	1.04 [0.99–1.09]	0.17
*Chronic malnutrition*						
o No	202 (86.0)	54 (78.3)	ref	0.13		
o Yes	33 (14.0)	15 (21.7)	1.70 [0.86–3.36]			
*Diabetes mellitus*						
o No	164 (69.2)	41 (59.4)	ref	0.13		
o Yes	73 (30.8)	28 (40.6)	1.53 [0.88–2.67]			
*Excessive alcohol consumption*						
o No	206 (87.3)	59 (85.5)	ref	0.61		
o Former	5 (2.1)	3 (4.3)	2.10 [0.49–9.02]			
o Current	25 (10.6)	7 (10.1)	0.98 [0.40–2.37]			
*Current smoking*						
o No	210 (89.4)	54 (78.3)	ref	0.019	ref	0.012
o Yes	25 (10.6)	15 (21.7)	2.33 [1.15–4.73]		2.98 [1.27–7.02]	
*ALMI (kg/m*^*2*^*)*						
o ≤ 5.5	34 (14.5)	26 (41.9)	4.27 [2.29–7.95]	< 0.001	3.72 [1.78–7.78]	0.001
o > 5.5	201 (85.5)	36 (58.1)	ref		ref	
Intact parathyroid hormone (ng/ml)**	46 [35—63]	53 [35–85]	1.11 [1.03–1.21]	0.010	1.15 [1.05–1.25]	0.003
**25 (OH) vitamin D level (ng/ml)**	26.3 ± 9.6	24.2 ± 10.2	0.98 [0.95–1.01]	0.12		
*Proton pomp inhibitors*						
o No	152 (64.7)	39 (60.0)	ref	0.49		
o Yes	83 (35.3)	26 (60.0)	1.22 [0.70–2.15]			

^*^ In case of history of bariatric surgery (*N* = 236). For patients without history of bariatric surgery, the time between last surgery and bone health assessment were considered at 0

^**^ Odds ratios and their 95% confidence intervals were estimated for the 10 ng/ml increase in intact parathyroid hormone

Values expressed as median [25th–75th percentiles], mean ± standard deviation or number (percentage). ALMI: appendicular lean mass index, AOM: anti-osteoporotic medication, CI: confidence interval, OR: odds ratio, ref: reference modality for the calculation of OR

### AOM Eligibility: Preoperative Versus Postoperative Assessment

Among the study population, 9 patients (13.0%) met AOM eligibility criteria preoperatively, compared with 60 patients (87.0%) postoperatively (Table 4).

## Discussion

One in five postmenopausal women was eligible for AOM (22.5%, *n* = 69/306) based on the ECTS position statement as part of their care pathway, either before or after MBS. This prevalence was significantly higher in women aged ≥ 60 years than in those aged < 60 years (30.1% vs. 16.8%, *p* = 0.006). Beyond the age of ≥ 60 years, factors such as active smoking, secondary hyperparathyroidism, and decreased ALMI were identified as independent risk factors.

Of the 306 postmenopausal women who underwent a bariatric surgery care pathway, 69 were assessed prior to their first surgery, 191 during follow-up, and 46 prior to a subsequent surgery. According to the 2022 ECTS criteria, 22.5% of the 306 postmenopausal women in our cohort were eligible for AOM, consistent with Courtalin et al*.*, who reported a 19.2% prevalence among 170 men over 50 and postmenopausal women, with no difference by surgical status [11]. Few studies have simultaneously assessed fractures and BMD after bariatric surgery. Blom-Høgestøl et al*.* followed 194 patients, including 59 postmenopausal women or men over 50, for 10 years after RYGB, finding osteopenia in 51%, osteoporosis in 27%, and 19% experiencing a low-energy fracture [12]. In comparison, almost 10% of our patients had sustained a fragility fracture within the preceding two years. However, Blom-Høgestøl et al*.* did not report the proportion of patients with a T-score ≤ –2, limiting direct comparison. Other studies focused on BMD loss: Lindeman et al*.* followed 21 patients up to five years after RYGB, observing significant decreases at the lumbar spine (−7.8%) and total hip (−15.3%) [13]. Schäfer et al. analyzed postmenopausal women separately and found greater preoperative vulnerability and more pronounced BMD loss at 12 months (total hip –12.2%) compared to premenopausal women (−7.2%) and men (−6.8%, *p* ≤ 0.02) [14]. Collectively, these findings confirm the heightened skeletal risk in postmenopausal women following MBS, although direct comparisons remain limited due to inconsistent reporting of T-scores ≤ -2.

While postoperative complications of MBS in patients over 60 have been well documented, few studies have specifically addressed bone health. The French HAS highlighted increased morbidity and mortality in older adults [3] but did not provide guidance on musculoskeletal assessment or fracture risk screening. An American study of 351,292 patients (12.6% aged > 65) reported a 3% postoperative mortality rate and higher rates of infectious, respiratory, and hospital-related complications among older adults [15]. Another study comparing RYGB and SG in patients ≥ 65 found frequent RYGB complications, including gastrointestinal ulcers (7.2%), anastomotic strictures (5.9%), and reinterventions (4.7%) [16]. However, no study has specifically examined bone-related consequences in this vulnerable group. Osteoporosis significantly contributes to morbidity and mortality, with one-year post-hip fracture mortality in older adults estimated at 15–30% [17], underscoring the need to evaluate long-term musculoskeletal outcomes in older postmenopausal women.

Beyond age, we aimed to identify risk factors, particularly modifiable ones, independently associated with eligibility for AOM, with the goal of targeting preventive strategies and better identifying high-risk populations.

Elevated iPTH emerged as an independent predictor of AOM eligibility, consistent with its role in bone resorption and skeletal fragility in secondary hyperparathyroidism [18, 19]. Supporting this, Wei et al*.* reported an increase in secondary hyperparathyroidism from 21% preoperatively to 35% one year after bariatric surgery (SG, RYGB, AG), primarily driven by vitamin D deficiency [20], a finding echoed in Saudi patients undergoing SG [21]. In our cohort, over 25% of patients had iPTH > 66 pg/mL, reflecting suboptimal vitamin D status (mean 25(OH)D: 25.8 ng/mL) and insufficient supplementation. French guidelines recommend maintaining 25(OH)D levels between 30–60 ng/mL, with monthly supplementation of 100,000 IU after RYGB and 50,000 IU after SG [22], highlighting a persistent gap between recommendations and real-world practice that underscores the need to optimize preventive strategies. Calcium absorption is compromised following both RYGB and SG. Consequently, even under conditions of optimal vitamin D status, PwO may not achieve adequate calcium absorption after bariatric surgery, which could constitute an additional mechanism underlying elevated iPTH levels.

Muscle parameters also significantly influence therapeutic decisions. Reduced ALMI (≤ 5.5 vs. > 5.5 kg/m^2^) was identified as an independent risk factor for initiating AOM, likely reflecting the loss of muscle’s mechanical and metabolic support for bone, which disrupts remodeling and decreases BMD [23]. Sarcopenia, particularly sarcopenic obesity, combines reduced muscle mass and strength with excess adiposity, impairing bone quality, increasing fall risk, and raising fracture incidence [24]. In postmenopausal women, lower appendicular muscle mass correlates with BMD loss at vulnerable sites such as the femoral neck, further elevating osteoporosis risk [25]. Tailored physical activity programs following MBS have been shown to mitigate postoperative bone loss, particularly in patients at risk of sarcopenic obesity [26, 27].

Physical inactivity is common among candidates for MBS, with fewer than 30% meeting the WHO-recommended 150 min of moderate weekly activity [28, 29]. Sedentary behavior, averaging over eight hours of sitting per day, exacerbates the metabolic and musculoskeletal consequences of severe obesity [30]. French guidelines stress that adequate protein intake, often insufficient in this population, is essential to preserve muscle mass and should complement regular physical activity [22].

Active smoking also emerged as a strong determinant of AOM eligibility, conferring a three-fold increased risk. This finding reinforces the well-established role of tobacco in accelerating bone loss and osteoporosis [31, 32], and is consistent with Fashandi et al*.*, who identified smoking as an independent predictor of fractures after MBS [33]. Collectively, these observations highlight the critical need to systematically integrate lifestyle interventions—including smoking cessation, structured physical activity, and adequate protein and calcium/vitamin D supplementation—into the pre- and postoperative management of postmenopausal women.

This study has several strengths. It was based on prospective inclusion with standardized and comprehensive data collection, including DXA scans, biochemical markers, MBS history, and clinical risk factors for osteoporosis. The sample size (*n* = 306) provided adequate power to identify factors associated with AOM eligibility. Over 95% of DXA scans were performed on the same device at Lille University Hospital, ensuring measurement consistency. Biochemical data, particularly vitamin D and iPTH levels, were available for over 90% of patients. Importantly, this is the first study to specifically investigate musculoskeletal health and independent risk factors for AOM eligibility in postmenopausal women aged ≥ 60 years undergoing bariatric surgery.

This study also has limitations. Its retrospective analysis may have introduced recall bias and missing data, particularly regarding fracture history or specific risk factors. Not all biochemical markers were systematically or recently assessed, potentially limiting interpretation, although overall data completeness remained high. Alcohol and smoking were self-reported, which may have led to underestimation. The cohort was heterogeneous, including both pre- and post-operative patients at various stages, reflecting real-world practice but introducing variability. Referral bias may have led to overrepresentation of postmenopausal bariatric women at higher fracture risk, potentially overestimating the prevalence of AOM eligibility. Finally, the monocentric design and the high proportion of previously operated patients (> 75%) may limit generalizability to other settings. Another limitation is the lack of a non-bariatric control group, limiting assessment of whether prevalence deviates from age-adjusted expectations.

Our findings emphasize the crucial importance of preventing and managing secondary osteoporosis both before and after MBS in postmenopausal women, particularly those aged 60 years and older. Beyond age, several independent risk factors were identified, underscoring the essential role of lifestyle interventions alongside AOM. Specifically, smoking cessation, minimizing sedentary behavior, and ensuring lifelong vitamin D and calcium supplementation to prevent secondary hyperparathyroidism should remain central priorities in both preoperative and postoperative care.
